# How locus of control shapes digital nomads’ subjective well-being: the mediating roles of telepressure, technostress, and boundary control

**DOI:** 10.1186/s40359-026-05635-7

**Published:** 2026-09-26

**Authors:** Qi Cao, ZiRan Zhou, ZiXuan Xu, YiMo Han, Hui-Ling Hu

**Affiliations:** 1 Graduate School of International Studies, Department of East Asian Studies, Hanyang University, Seoul, 04763 Republic of Korea; 2 School of Humanities and Social Science, The Hong Kong University of Science and Technology, Clear Water Bay, Kowloon, Hong Kong SAR, China; 3 Department of Mathematics, Faculty of Science, National University of Singapore, Singapore, 119077 Singapore; 4 School of Business, Yangzhou University, Yangzhou, 225009 China; 5 Department of Social and Behavioural Sciences, City University of Hong Kong, Hong Kong SAR, China; 6 Institute of Creative Design and Management, National Taipei University of Business, Taoyuan, 324022 Taiwan

**Keywords:** Digital nomads, Locus of control, Telepressure, Technostress, Boundary control, Subjective well-being, Offline social interaction

## Abstract

**Background:**

As digital nomadism becomes an increasingly prevalent form of digital labor, understanding how psychological mechanisms shape well-being under highly autonomous yet weakly institutionalized work conditions is critical. Drawing on the Job Demands–Resources (JD-R) model and Conservation of Resources (COR) theory, this study examines how locus of control influences digital nomads’ subjective well-being through technostress, telepressure, and boundary control, while also testing the moderating role of offline social interaction.

**Methods:**

Survey data from 540 digital nomads recruited through online remote work and digital nomad communities were analyzed using partial least squares structural equation modeling (PLS-SEM) with a bootstrapping procedure based on 5,000 resamples.

**Results:**

The results show that locus of control does not directly affect subjective well-being but operates through multiple mediating pathways. A stronger internal locus of control reduces technostress and telepressure and enhances boundary control, which in turn improves subjective well-being. Boundary control emerges as the strongest positive predictor of well-being. In addition, offline social interaction significantly buffers the negative effect of telepressure on subjective well-being.

**Conclusions:**

These findings suggest that digital nomads’ well-being depends less on work flexibility itself and more on individuals’ capacity to manage digital demands and psychological boundaries.

**Supplementary Information:**

The online version contains supplementary material available at https://doi.org/10.1186/s40359-026-05635-7.

## Introduction

With the rapid maturation of information and communication technologies (ICTs), the traditional linkage between work and place has gradually disintegrated. Digital nomads—an emerging form of labor that combines remote work with a highly mobile lifestyle—have become an important yet still insufficiently understood group within the contemporary digital economy [1, 2]. Compared with traditional organizational employees, digital nomads exhibit a highly decentralized configuration in terms of time, space, and organizational affiliation. Their work typically relies on digital platforms, cross-time-zone communication, and multiple technological tools, while responsibility for work pacing, boundary management, and life-resource integration rests largely on the individual. Although this work arrangement is often portrayed as a symbol of high autonomy and lifestyle freedom, recent studies have increasingly indicated that it also entails substantial psychological strain and risks to subjective well-being [3, 4].

Particularly within remote and digitally mediated work contexts, autonomy is frequently accompanied by a paradoxical phenomenon known as the *autonomy paradox*. Technologies designed to enhance flexibility and efficiency simultaneously intensify expectations of immediate responsiveness, constant availability, and role blurring, thereby undermining psychological recovery and well-being [5]. For digital nomads, this paradox is especially pronounced, as income stability and project continuity often depend on rapid responses, online visibility, and cross-time-zone coordination. As a result, “being always available” is gradually internalized from an external requirement into a form of psychological pressure.

Barber and Santuzzi, [6] define telepressure as a preoccupation with work-related messages and a compulsive urge to respond immediately. Subsequent research has demonstrated that telepressure is consistently associated with poorer sleep quality, increased psychological exhaustion, and reduced well-being [7]. Telepressure may therefore be a key mechanism undermining subjective well-being (SWB) under the asynchronous, boundary-spanning conditions of nomadic work.

Digital nomads are also continuously exposed to multiple tools and platforms, and hence to technostress, whose dimensions of techno-overload, techno-invasion, techno-complexity, and techno-uncertainty can trigger anxiety, loss of control, and exhaustion [8, 9]. Research on remote work further shows that technostress significantly reduces job satisfaction and overall well-being [4, 10]. However, existing studies have largely focused on employees within formal organizational settings. How technostress translates into well-being outcomes among highly mobile digital nomads—who lack stable organizational support—remains insufficiently examined.

To better understand the well-being mechanisms of digital nomads in highly digitalized contexts, this study adopts the Job Demands–Resources (JD-R) model and Conservation of Resources (COR) theory as an integrative theoretical framework. In this framework, demands consume psychological energy while resources enable recovery [11]. COR theory adds that stress arises from threatened or actual resource loss, which can develop into a loss spiral [12]. Within the digital nomad context, telepressure and technostress can be understood as core job demands, whereas the ability to effectively manage work–life boundaries constitutes a critical psychological resource.

According to boundary theory, when structural boundaries between work and non-work roles weaken, individuals’ perceived control over boundary management becomes a decisive factor influencing role conflict and well-being [13]. Kossek et al., [14] conceptualize boundary control as individuals’ perceived ability to determine the timing, manner, and intensity of role transitions. Empirical evidence suggests that boundary control mitigates work–life conflict and enhances psychological well-being in remote work contexts [15].

Stress experiences and boundary management nonetheless differ markedly across digital nomads, which points to a deeper individual-leve l foundation. This study argues that *locus of control* serves as a critical theoretical link connecting digital work demands to well-being. Originating from Rotter’s [16] social learning theory, locus of control refers to individuals’ tendencies to attribute outcomes to their own actions (internal control) or to external forces (external control). Extensive research indicates that an internal locus of control is associated with stronger self-regulation, lower perceived stress, and better psychological health [17, 18]. In the uncertain and self-managed context of digital nomadism, individuals with a stronger internal locus of control are more likely to perceive technological demands as manageable challenges rather than uncontrollable threats, thereby reducing technostress and telepressure and enhancing boundary control.

In addition, this study introduces offline social interaction as a key contextual moderator. Although digital nomads can maintain connections through online communities, research indicates that the absence of stable and meaningful face-to-face interactions may still lead to loneliness and psychological exhaustion [19]. Offline social interaction may both buffer the negative effects of stress on well-being and strengthen the positive impact of boundary control on subjective well-being [20].

Taken together, this study proposes an integrative model in which locus of control serves as a core antecedent influencing subjective well-being through multiple mediating mechanisms—telepressure, technostress, and boundary control—while offline social interaction operates as a moderating condition. This framework addresses three key theoretical gaps: first, it extends understanding of how telepressure and technostress affect well-being in the digital nomad context; second, it translates locus of control into concrete psychological mechanisms of digital stress and boundary governance; and third, it elucidates how offline social interaction provides protective and amplifying effects in highly digitalized lifestyles.

## Literature review

### Digital nomadism as an extreme digital work context

The rapid diffusion of information and communication technologies (ICTs) has fundamentally reshaped the relationship between work, time, and place. Beyond conventional telecommuting or hybrid work arrangements, *digital nomadism* has emerged as a distinctive form of work characterized by location independence, high geographic mobility, and intensive reliance on digital technologies [1, 21]. Digital nomads typically perform their work remotely while continuously moving across cities or countries, relying on digital platforms, cloud-based collaboration tools, and mobile connectivity to sustain their livelihoods.

Compared with conventional remote workers, digital nomads operate under markedly different structural conditions. While many remote employees remain embedded within formal organizations—benefiting from stable contracts, managerial oversight, and institutionalized support systems—digital nomads often work as freelancers, independent contractors, or platform-based workers with limited organizational affiliation [2]. Their employment relationships are frequently short-term, project-based, and mediated by digital platforms, resulting in weaker organizational boundaries and reduced access to formal resources such as human resource support, clear performance norms, or regulated working hours. This structural detachment shifts responsibility for work scheduling, performance regulation, and recovery almost entirely onto the individual.

From a work design perspective, digital nomadism can therefore be conceptualized as an extreme digital work context, one in which autonomy is high but institutional scaffolding is minimal. Mazmanian et al., [5] describe this phenomenon as the autonomy paradox, whereby technologies intended to enhance flexibility simultaneously intensify expectations of constant availability, responsiveness, and self-monitoring. In the absence of organizational boundaries, digital nomads must continuously negotiate when to work, where to work, and how to remain visible and responsive to clients or collaborators across time zones.

Digital nomads typically rely on multiple communication platforms, productivity applications, and online marketplaces to secure work, coordinate tasks, and maintain professional relationships. This technological intensity increases exposure to information overload, rapid technological change, and continual connectivity, all of which have been identified as key antecedents of technostress in digital work environments [8, 22].

At the same time, digital nomadism is characterized by high boundary permeability between work and non-work domains. Work is frequently performed in cafés, co-working spaces, or temporary accommodations, blurring spatial and temporal boundaries that traditionally separate professional and personal roles. Boundary theory suggests that when structural boundaries are weak, individuals become more vulnerable to role intrusion and conflict, particularly when expectations of availability are ambiguous or unregulated [13]. Empirical studies on digital nomads indicate that the absence of stable routines and fixed workspaces may intensify difficulties in maintaining work–life boundaries, despite the appearance of flexibility [23].

Taken together, digital nomadism combines three characteristics that render it theoretically significant for research on digital work and well-being: (1) high self-management demands due to weak organizational embeddedness, (2) intensive reliance on digital technologies as the primary means of work coordination, and (3) heightened boundary permeability across time, space, and roles. These conditions make digital nomads particularly susceptible to digitally induced job demands, such as technostress and telepressure, while simultaneously increasing the importance of personal resources that enable boundary regulation and psychological recovery.

These conditions do more than intensify the demands documented in the remote work literature; they alter the mechanism through which demands and resources are converted into well-being outcomes, and two conditions in particular are pivotal. First, the absence of a formal organizational structure removes what Petriglieri et al., [24] describe as the holding environment that ordinarily absorbs occupational uncertainty on the worker’s behalf. For organizational teleworkers, supervisory feedback, codified performance norms, and human resource systems supply external referents against which effort and availability can be calibrated [25]. Digital nomads possess no such referents, so the judgment of what constitutes a legitimate boundary rests wholly on the individual. Locus of control therefore ceases to be one determinant of boundary regulation among several and becomes its principal governing mechanism—an argument that does not extend to salaried teleworkers, whose boundaries are partly set for them. Second, income instability changes the nature of telepressure rather than its magnitude. In salaried telework, the urge to respond immediately is driven largely by social and normative expectations of availability [6]. For platform-mediated and freelance nomads, responsiveness is directly priced: response times, online visibility, and client ratings feed reputation systems that govern future work allocation [26, 27]. Telepressure is thereby converted from a normative demand into an economic survival demand, which in JD-R terms means it is no longer amenable to the resources that ordinarily buffer normative pressure. It is this qualitative reconfiguration, rather than a higher dose of the same demands, that warrants treating digital nomadism as a theoretically distinct context instead of a subset of remote or platform work.

### Locus of control as a core psychological resource in digital work

Locus of control, originally proposed within Rotter’s [16] social learning theory, refers to individuals’ generalized beliefs regarding the extent to which life outcomes are contingent upon their own actions versus external forces. Individuals with an internal locus of control tend to believe that outcomes are primarily shaped by personal effort, competence, and decision-making, whereas those with an external locus of control are more likely to attribute outcomes to chance, powerful others, or situational constraints. Beyond shaping motivational orientations, locus of control has been shown to play a critical role in individuals’ cognitive appraisals of stressors and their coping responses.

A substantial body of empirical research demonstrates that an internal locus of control is associated with a range of positive psychological and behavioral outcomes. Meta-analytic evidence indicates that individuals with stronger internal control beliefs report higher job satisfaction, better job performance, and lower perceived stress [18]. Longitudinal research in health psychology further suggests that internal locus of control predicts better psychological health and higher subjective well-being across the life course [17]. Collectively, these findings position locus of control as a relatively stable individual difference that exerts systematic influence on how individuals perceive and respond to demanding environments.

From the perspective of Conservation of Resources (COR) theory, perceived control constitutes a key personal resource that enables individuals to protect existing resources and invest in new ones [12]. When individuals believe they can influence outcomes through their own actions, they are less likely to interpret environmental demands as threatening and more likely to engage in proactive coping behaviors. Conversely, when outcomes are perceived as largely uncontrollable, individuals are more vulnerable to stress reactions and resource loss spirals. In this sense, locus of control functions not merely as a personality trait, but as a psychological resource that shapes how demands translate into strain.

Within the context of digital nomadism—conceptualized in this study as an extreme digital work context—locus of control assumes heightened importance. Digital nomads typically operate with limited organizational embeddedness and weak institutional support, placing primary responsibility for work scheduling, technology management, and boundary regulation on the individual. In the absence of formal structures, individuals with a stronger internal locus of control are more likely to perceive digital work demands as manageable challenges and to enact self-regulatory strategies that mitigate stress. In contrast, individuals with a stronger external locus of control may experience digital demands as externally imposed and difficult to escape, increasing their susceptibility to psychological strain.

The present study therefore positions locus of control as a core antecedent that influences technostress, telepressure, and boundary control, thereby setting the stage for subsequent hypotheses regarding its indirect effects on subjective well-being.

### Locus of control, technostress, and telepressure

#### Locus of control and technostress

Technostress refers to the stress experienced by individuals as a result of using information and communication technologies, typically arising from techno-overload, techno-invasion, techno-complexity, and techno-uncertainty [8]. In highly digitalized work environments, technologies no longer merely support work activities but function as central infrastructures through which tasks, coordination, and performance expectations are enacted, thereby increasing individuals’ exposure to continuous connectivity and information intensity.

Prior research suggests that technostress is not determined solely by the objective extent of technology use, but is strongly shaped by individuals’ subjective interpretations of technological demands and their coping capabilities [22]. For example, the use of AI in hiring processes may reduce applicants’ trust, a reaction stemming from users’ inability to exert control over AI-driven selection procedures [28]. This perspective aligns closely with locus of control theory. Individuals with a stronger internal locus of control tend to perceive technological demands as manageable and adjustable challenges, and are more likely to adopt proactive coping strategies, such as optimizing workflows, filtering information, or redefining technology use practices. In contrast, individuals with a stronger external locus of control are more inclined to view technological demands as externally imposed and uncontrollable, thereby amplifying the stress associated with techno-overload and techno-invasion.

When technology is perceived as an uncontrollable force, stress reactions intensify and resource loss processes are more likely to occur [12]. In the digital nomad context, where formal technical support and standardized systems are largely absent, responsibility for managing technological complexity rests almost entirely on the individual, rendering locus of control particularly salient in shaping technostress experiences.

Accordingly, this study proposes that a stronger internal locus of control is associated with lower levels of technostress.

H1: Locus of control is negatively associated with technostress.

#### Locus of control and telepressure

Telepressure refers to the extent to which individuals are psychologically preoccupied with work-related messages and experience a compulsive urge to respond immediately. Its defining feature lies not in message volume, but in the internalization of response obligations and availability expectations [6].

In digital work contexts, telepressure is particularly salient when work boundaries are weak and performance outcomes depend heavily on responsiveness. For digital nomads, cross-time-zone collaboration, platform-based reputation systems, and project uncertainty often render rapid responsiveness an implicit norm. Whether such norms are experienced as unavoidable external pressures or as manageable behavioral choices depends largely on individuals’ control beliefs.

Individuals with a stronger internal locus of control are more likely to believe that they can regulate their availability and response rhythms, treating immediate responsiveness as a strategic decision rather than an obligatory demand. In contrast, individuals with a stronger external locus of control are more prone to internalize availability expectations as external pressures, making psychological detachment from work more difficult. Prior research has demonstrated that telepressure is associated with impaired psychological detachment, poorer sleep quality, and increased emotional exhaustion [7], underscoring its role as a key psychological stressor.

Accordingly, this study proposes that locus of control plays a crucial role in shaping individuals’ experiences of telepressure.

H2: Locus of control is negatively associated with telepressure.

### Locus of control and boundary control

Boundary theory posits that when structural boundaries—such as fixed working hours and physical workplaces—are weakened, the risk of role intrusion and role conflict increases, rendering individuals’ perceived ability to control boundaries a critical determinant of psychological adjustment and well-being [13].

Kossek et al., [14] conceptualize boundary control as individuals’ perceived ability to determine the timing, manner, and extent of transitions between work and non-work roles. Unlike boundary preferences (e.g., integration versus segmentation), boundary control emphasizes whether individuals can effectively enact boundary management strategies in line with their intentions. Empirical research has shown that higher levels of boundary control are associated with lower work–life conflict, enhanced psychological recovery, and improved well-being outcomes [15].

The importance of boundary control is particularly salient in digitalized work contexts characterized by weak organizational support. Remote and platform-based work arrangements increase temporal and spatial flexibility but simultaneously obscure availability norms and expectations. Digital nomads frequently work in cafés, co-working spaces, or temporary accommodations, lacking clear end-of-work cues or stable workspaces, which heightens the permeability of work–life boundaries [23]. Under such conditions, the ability to proactively establish and maintain boundaries becomes a crucial psychological resource.

Locus of control plays a pivotal role in shaping individuals’ capacity for boundary control. Individuals with a stronger internal locus of control are more likely to believe that they can influence their work–life arrangements and are therefore more inclined to actively implement boundary management strategies, such as regulating response windows, limiting availability, or structuring temporal and spatial routines. In contrast, individuals with a stronger external locus of control may perceive boundary management as constrained by external expectations or situational forces, resulting in lower perceived boundary control.

Based on these theoretical arguments, the following hypothesis is proposed:

H3: Locus of control is positively associated with boundary control.

### Technostress, telepressure, boundary control, and subjective well-being

Subjective well-being (SWB) is commonly defined as individuals’ overall evaluation of their quality of life, encompassing both cognitive judgments of life satisfaction and affective experiences of positive and negative emotions [29]. Compared with work-specific outcomes such as job satisfaction or organizational commitment, SWB captures a broader assessment of individuals’ psychological functioning, making it particularly suitable for examining the experiences of digital nomads operating in highly digitalized and weakly institutionalized work contexts.

Research on technostress has consistently shown that prolonged exposure to techno-overload, techno-invasion, and techno-uncertainty increases anxiety, emotional exhaustion, and burnout, ultimately undermining overall well-being [22]. Empirical studies in remote and digital work contexts further demonstrate that technostress is negatively associated with life satisfaction and psychological health [10]. For digital nomads, technologies are not merely tools for task execution but essential infrastructures for securing income and maintaining professional visibility, which makes detachment from technological demands particularly challenging and amplifies their detrimental effects on subjective well-being.

H4: Technostress is negatively associated with subjective well-being.

Prior research has demonstrated that telepressure is associated with impaired psychological detachment, poorer sleep quality, and reduced well-being [7]. In the context of digital nomadism, where cross-time-zone collaboration and implicit availability norms prevail, telepressure may function as a chronic stressor that persistently erodes individuals’ subjective well-being.

H5: Telepressure is negatively associated with subjective well-being.

In contrast to these digital job demands, boundary control represents a key psychological resource. Individuals who perceive greater control over transitions between work and non-work roles are better able to facilitate psychological detachment, reduce role conflict, and enhance overall well-being [15]. Boundary control restores a sense of autonomy and order, thereby supporting positive affective experiences and life satisfaction.

Boundary control is particularly consequential for digital nomads, whose work lacks fixed temporal and spatial boundaries. When effective boundary control is absent, digital nomads are more likely to experience prolonged working hours and cognitive overload. Conversely, those with higher boundary control are better equipped to maintain psychological balance within flexible work arrangements, leading to higher levels of subjective well-being. Empirical evidence from remote and flexible work research supports a positive association between boundary control and well-being outcomes [14].

H6: Boundary control is positively associated with subjective well-being.

In sum, technostress and telepressure function as digital job demands that undermine subjective well-being through resource depletion, whereas boundary control operates as a critical psychological resource that promotes recovery and well-being. This demand–resource configuration provides the theoretical foundation for subsequent analyses of multiple mediation and moderation mechanisms.

### Multiple mediation effects: locus of control, digital job demands/resources, and subjective well-being

The influence of individual characteristics on well-being is largely indirect, and especially so in highly digitalized and weakly institutionalized work environments, where personal attributes are more likely to influence well-being indirectly by shaping individuals’ experiences of job demands and their access to psychological resources [11].

Integrating the Job Demands–Resources (JD-R) model with Conservation of Resources (COR) theory, the present study proposes a multiple mediation framework that simultaneously incorporates demand-reduction and resource-enhancement pathways. Within COR theory, resource acquisition and protection enhance well-being [12].

Specifically, locus of control is expected to shape how individuals interpret digital job demands and mobilize psychological resources, thereby activating two complementary mediation pathways.

The first pathway is a demand-depletion pathway. Individuals with a stronger internal locus of control are less likely to internalize technological demands and availability expectations as uncontrollable external pressures, resulting in lower levels of technostress and telepressure. Reduced digital job demands, in turn, preserve attentional and emotional resources, thereby contributing to higher levels of subjective well-being. This pathway reflects the protective function of locus of control through the attenuation of digital job demands.

The second pathway is a resource-gain pathway. Individuals with a stronger internal locus of control are more inclined to believe that they can actively shape their work–life arrangements, which enhances their capacity to establish and utilize boundary control as a psychological resource. Higher boundary control facilitates psychological detachment, reduces role conflict, and restores a sense of autonomy and order, ultimately promoting subjective well-being. This pathway reflects the enabling function of locus of control through the enhancement of personal resources.

Importantly, these two pathways are theoretically complementary rather than mutually exclusive. Locus of control simultaneously reduces resource depletion by mitigating digital job demands and promotes resource accumulation by strengthening boundary control. Examining only a single mediator would therefore risk underestimating the overall influence of locus of control.

Based on these theoretical arguments, the following multiple mediation hypotheses are proposed:

H7: Locus of control indirectly enhances subjective well-being through reduced technostress.

H8: Locus of control indirectly enhances subjective well-being through reduced telepressure.

H9: Locus of control indirectly enhances subjective well-being through enhanced boundary control.

### The moderating role of offline social interaction

In highly digitalized and weakly institutionalized work contexts, the form and quality of social interaction constitute critical contextual factors shaping individuals’ psychological adjustment. Although digital technologies facilitate online communication and virtual communities, prior research suggests that offline (face-to-face) social interaction plays a unique role in providing emotional support, fostering psychological recovery, and cultivating a sense of belonging, functions that are not fully substitutable by online interaction [30]. For digital nomads, who often experience prolonged mobility and detachment from stable social networks, offline social interaction may represent a particularly important contextual resource.

From a Conservation of Resources (COR) perspective, social support is a key external resource that replenishes depleted psychological energy and mitigates resource loss spirals under stress [12]. Similarly, the Job Demands–Resources (JD-R) model posits that social support operates as a contextual resource that buffers the adverse effects of job demands on psychological outcomes [11]. Within this framework, offline social interaction is expected to exert not only direct benefits for well-being but also moderating effects on how digital job demands and psychological resources translate into subjective well-being.

Two features of the digital nomad context make offline interaction specifically—rather than social support in general—the theoretically appropriate moderator. First, because digital nomads’ work, coordination, and professional identity are all enacted through the same digital channels, online social contact does not constitute a genuine change of context; it re-enters the very medium from which the demand originates and therefore affords little psychological detachment [31]. Offline, co-present interaction changes the medium as well as the content of interaction, and it is this discontinuity, rather than support per se, that permits recovery. Second, COR theory holds that resources travel in caravans sustained by passageways, that is, by the stable environments and relationships that generate and protect them [12]. Sustained mobility systematically erodes such passageways for digital nomads: relationships are repeatedly interrupted by relocation, and the organizational holding environment that ordinarily supplies identity confirmation is absent [24]. Offline interaction in co-working spaces, nomad hubs, and local communities operates as a substitute passageway, furnishing the embodied recognition that online exchange conveys only weakly. Offline social interaction is therefore theorized to moderate not because it adds a further increment of support, but because it restores the contextual conditions under which personal resources can be replenished and deployed.

First, offline social interaction is expected to buffer the negative effect of technostress on subjective well-being. Technostress continuously consumes attentional resources and induces cognitive fatigue, whereas face-to-face social interaction facilitates emotional expression, attentional disengagement from work, and psychological recovery [31]. When digital nomads engage in high-quality offline social interactions, emotional support and re-anchoring in non-work roles may attenuate the detrimental impact of technostress on overall well-being.

H10: Offline social interaction weakens the negative relationship between technostress and subjective well-being.

Second, offline social interaction may also buffer the negative effect of telepressure on subjective well-being. Telepressure reflects psychological preoccupation with work-related messages and urges for immediate responsiveness. Offline social interactions are typically immersive and socially engaging, thereby facilitating psychological detachment and role transitions. Prior research indicates that engagement in meaningful non-work social activities reduces work-related rumination and protects well-being [20]. Accordingly, offline social interaction may function as a contextual barrier that diminishes the adverse effects of telepressure on subjective well-being.

H11: Offline social interaction weakens the negative relationship between telepressure and subjective well-being.

Finally, offline social interaction is expected to strengthen the positive relationship between boundary control and subjective well-being. Because boundary control confers the capacity to disengage, its value for well-being depends on the existence of non-work roles worth protecting. Boundary theory further holds that role transitions are enacted and validated socially, so a boundary that no one else recognizes is costly to sustain [13]. Stable offline relationships supply both the destination and the social validation for such transitions, converting the capacity to disengage into genuinely restorative experience. In contrast, when offline social interaction is limited, the positive effects of boundary control may be constrained by a lack of meaningful alternatives to work engagement.

H12: Offline social interaction strengthens the positive relationship between boundary control and subjective well-being.

In sum, offline social interaction plays a dual moderating role in digital work contexts: it buffers the negative effects of digital job demands on well-being while amplifying the positive effects of psychological resources. Incorporating offline social interaction as a moderator enables a more nuanced understanding of the contextual conditions under which digital nomads’ subjective well-being is shaped.

**Fig. 1:**
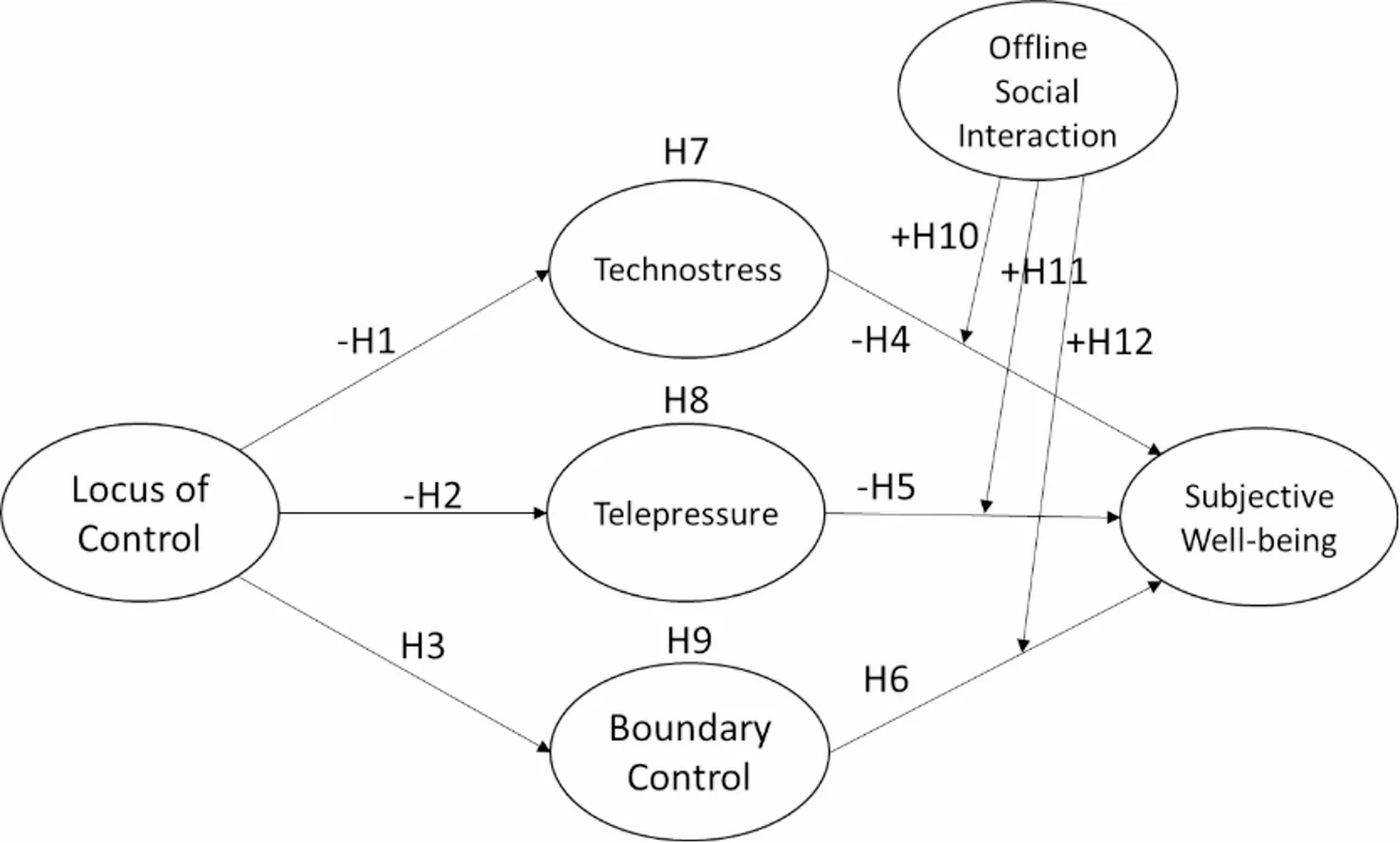
Research framework

This study proposes the research framework as illustrated in Fig. 1.

## Methodology

### Research participants and data collection

This study targeted digital nomads as the research population, defined as professional workers who rely heavily on digital technologies to perform location-independent or geographically flexible work. Data were collected between November and December 2025 through an online questionnaire hosted on Wenjuanxing (Sojump), a survey platform widely used in Chinese-speaking regions. Recruitment was community-based: invitations containing the survey link were posted in remote work communities, digital nomad forums, and social media groups whose membership is defined by location-independent working. Because the link was circulated openly within these communities rather than issued to a closed panel, the number of individuals who viewed the invitation is unknown and a response rate cannot be computed. Participation was voluntary and unpaid, and completion required approximately 15 to 20 min. A total of 540 valid questionnaires were obtained for analysis. Eligibility screening was administered by the survey platform, which was instructed to admit only respondents with actual digital nomad work experience. All participants additionally reported performing paid work remotely and reported their weekly remote working hours, which were retained as a control variable. Because the platform’s screening procedure is proprietary, its precise operational criteria were not disclosed to the research team, and no additional screening or attention-check items were embedded in the questionnaire itself; the implications of this are considered in the Limitations and future research directions section. Questionnaires with incomplete responses or evidently invalid patterns were excluded. Participants provided informed consent on the opening page of the questionnaire before proceeding, no identifiable personal information was recorded, and all data were used solely for academic research purposes in compliance with established research ethics guidelines.

### Measurement instruments

This study employed a structured questionnaire to collect data. All constructs were measured using a seven-point Likert scale ranging from 1 (strongly disagree) to 7 (strongly agree). The final instrument comprised 34 items across six constructs, preceded by a demographic section. With the exception of offline social interaction, the measurement items were adapted from well-established scales in prior literature and were contextually modified to reflect the work and lifestyle characteristics of digital nomads. Such adaptation ensures both theoretical consistency with prior research and contextual relevance to the digital nomad setting.

Locus of control was grounded in Rotter’s [16] social learning theory and refers to individuals’ beliefs regarding whether life outcomes are contingent upon their own actions and abilities. Prior research has shown that in highly autonomous and flexible work environments, locus of control plays a significant role in shaping individuals’ interpretations of stress and their psychological adjustment capabilities [18]. Internal locus of control was measured with six items capturing perceived control over work outcomes, work decisions, and the proactive creation of opportunities (e.g., “I can influence my work outcomes”).

Telepressure refers to the psychological pressure and tension associated with the perceived need to respond immediately to work-related messages enabled by communication technologies [6]. Six items assessed the urge to reply promptly, attentional disruption caused by incoming messages, and the discomfort arising from delayed responses (e.g., “I feel a strong need to respond to others immediately”). These items were designed to reflect the prolonged connectivity and cross-time-zone interactions characteristic of digital nomadic work.

Technostress was operationalized based on the framework proposed by Tarafdar et al., [32], encompassing three dimensions: techno-overload, techno-invasion, and techno-complexity. Nine items were used, three for each dimension (e.g., techno-overload: “Technology requires me to do more work in less time”; techno-invasion: “Technology brings work into my private life”; techno-complexity: “New tools often confuse me”). The items captured the extent to which digital technologies impose pressure on work pace, intrude into personal life boundaries, and consume cognitive resources, reflecting the psychological burden associated with continuous adaptation to new tools and platforms in digital work environments.

Boundary control, derived from work–life boundary theory [13], refers to individuals’ perceived ability to establish and maintain boundaries between different life roles. In this study, boundary control was measured by focusing on respondents’ autonomy in managing response timing, notification settings, and personal life boundaries, thereby assessing their capacity to regulate technological demands and maintain psychological balance in highly connected environments.

Offline social interaction was measured with four items developed for this study, the content of which was guided by two established conceptualizations: the frequency-and-affect account of informal, non-task-related social interaction at work [33], and the perceived emotional support dimension of social support [34]. The items assess the frequency of face-to-face contact with friends and colleagues and the emotional support and sense of connectedness derived from it (e.g., “I receive emotional support from in-person interactions”). This construct was conceptualized as an important social resource that buffers technostress and facilitates psychological recovery, particularly for individuals engaged in long-term remote and highly digitalized work arrangements.

Finally, subjective well-being was used to assess individuals’ overall psychological state, encompassing positive affect, vitality, and life satisfaction. This construct was theoretically informed by Diener et al., [35] and the WHO-5 Well-Being Index and has been widely applied in research on mental health and work-related well-being. Five items assessed positive affect, calmness, vitality, restedness, and interest in daily activities (e.g., “I feel cheerful and in good spirits”).

In addition, several demographic variables were collected as control variables, including gender, age, educational attainment, occupational category, weekly remote working hours, and current city of residence. Incorporating these variables helps control for potential confounding effects of individual background characteristics on psychological states and technology use experiences, thereby enhancing the robustness and explanatory power of the research findings.

### Data analysis method

This study employed Partial Least Squares Structural Equation Modeling (PLS-SEM) as the primary data analysis technique, using SmartPLS 4.0 for model estimation and hypothesis testing. PLS-SEM is particularly appropriate for theory-driven predictive models and complex structural models involving multiple mediation and moderation effects [36]. Prior to structural model evaluation, the measurement model was assessed to ensure adequate reliability and validity. Specifically, indicator reliability, internal consistency reliability (Composite Reliability), convergent validity (Average Variance Extracted, AVE), and discriminant validity were examined. Discriminant validity was evaluated using the Fornell–Larcker criterion.

Regarding sample size adequacy, a total of 540 valid questionnaires were included in the final analysis, meeting the methodological requirements for PLS-SEM. According to the ten-times rule, the minimum sample size should be at least ten times the maximum number of structural paths pointing to any single latent variable [37]. In the present model, no endogenous construct received more than four structural paths, resulting in a minimum required sample size of approximately 40 observations.

In addition, statistical power considerations indicate that when the sample size exceeds 200, structural equation models are generally capable of detecting medium effect sizes (f² ≈ 0.15) with adequate statistical power (power ≥ 0.80) at a significance level of α = 0.05 [38, 39]. Therefore, the sample size employed in this study not only exceeds the minimum estimation threshold but also provides sufficient power for the robust testing of multiple mediation and moderation effects.

For structural model evaluation, hypothesis testing was conducted by examining path coefficients and coefficients of determination (*R*²). A bootstrapping procedure with 5,000 resamples was applied to estimate the significance levels and confidence intervals of the model parameters. Furthermore, indirect effect analyses were performed to test the multiple mediation effects of technostress, telepressure, and boundary control, while interaction effect analyses were conducted to assess the moderating role of offline social interaction. These procedures enabled a comprehensive examination of the theoretical hypotheses proposed in this study.

## Research results

### Demographic characteristics of the respondents

Table 1 presents the demographic characteristics of the respondents. Among the 540 valid participants, females constituted the majority of the sample (70.7%), while males accounted for 29.3%. Regarding age distribution, the largest proportion of respondents were aged 40–49 years (30.0%), followed by those aged 18–24 years (26.3%). In terms of educational attainment, more than half of the respondents held a bachelor’s degree (55.0%), whereas 16.5% had obtained a master’s degree or higher. Concerning weekly remote working hours over the past month, the majority of respondents reported working remotely for 0–10 h per week (64.3%), indicating that most participants engaged in relatively limited remote work during the study period.

**Table 1:** Demographic characteristics of the respondents (*N* = 540)

Category	Group	Frequency	Percentage (%)
Gender	Male	158	29.3
	Female	382	70.7
Age	18–24	142	26.3
	25–29	67	12.4
	30–34	38	7.0
	35–39	50	9.3
	40–49	162	30.0
	50 years or above	81	15.0
Education	High school or below	67	12.4
	Associate degree	87	16.1
	Bachelor’s degree	297	55.0
	Master’s degree or above	89	16.5
Weekly Remote Working Hours (Past Month)	0–10 h	347	64.3
	11–19 h	52	9.6
	20–29 h	40	7.4
	30–39 h	35	6.5
	40–49 h	31	5.7
	50 h or more	35	6.5

### Convergent validity

Table 2 presents the results of the reliability and convergent validity assessment of the measurement model. Following established guidelines for Partial Least Squares Structural Equation Modeling (PLS-SEM), indicator reliability was first evaluated by examining outer loadings, with values above 0.70 indicating adequate item reliability [37]. As shown in Table 2, all measurement items exhibited satisfactory outer loadings exceeding the recommended threshold, thereby supporting indicator reliability. Internal consistency reliability was assessed using Cronbach’s alpha and composite reliability (CR). All constructs demonstrated Cronbach’s alpha values greater than 0.70, indicating acceptable internal consistency. In addition, the CR values for all constructs exceeded the recommended minimum of 0.70, further confirming the reliability of the measurement scales. Convergent validity was evaluated using the average variance extracted (AVE). The AVE values for all constructs were above the threshold of 0.50, suggesting that each construct explains more than half of the variance of its indicators. Taken together, these results provide strong evidence of adequate reliability and convergent validity for the measurement model, supporting its suitability for subsequent structural model analysis.

**Table 2:** Reliability and convergent validity of the measurement model

Construct	Item	Outer loadings	Cronbach’s alpha	Composite reliability	Average variance extracted (AVE)
Boundary Control	BC1	0.838	0.871	0.912	0.723
	BC2	0.882			
	BC3	0.893			
	BC4	0.785			
Locus of Control	LOC1	0.743	0.906	0.927	0.681
	LOC2	0.780			
	LOC3	0.828			
	LOC4	0.848			
	LOC5	0.887			
	LOC6	0.858			
Offline Social Interaction	OSI1	0.839	0.863	0.907	0.710
	OSI2	0.765			
	OSI3	0.887			
	OSI4	0.874			
Subjective Well-being	SWB1	0.910	0.942	0.956	0.812
	SWB2	0.917			
	SWB3	0.917			
	SWB4	0.875			
	SWB5	0.886			
Telepressure	TE1	0.765	0.887	0.913	0.636
	TE2	0.813			
	TE3	0.827			
	TE4	0.818			
	TE5	0.761			
	TE6	0.798			
Techno-Complexity	TSCX1	0.880	0.889	0.931	0.819
	TSCX2	0.919			
	TSCX3	0.915			
Techno-Invasion	TSIV1	0.848	0.843	0.905	0.762
	TSIV2	0.891			
	TSIV3	0.879			
Techno-overload	TSOL1	0.778	0.718	0.842	0.639
	TSOL2	0.830			
	TSOL3	0.790			
Technostress	TSCX	0.818	0.789	0.877	0.704
	TSIV	0.869			
	TSOL	0.829			

### Discriminant validity

Discriminant validity was assessed using the Fornell–Larcker criterion [40]. According to this criterion, discriminant validity is established when the square root of the average variance extracted (AVE) for each latent construct is greater than its correlations with other constructs. This approach has been widely applied in structural equation modeling research as a classical and representative statistical standard for evaluating the conceptual distinctiveness of latent variables. Subsequent methodological studies have further indicated that the Fornell–Larcker criterion remains appropriate and informative for multidimensional models in which constructs are theoretically related yet conceptually distinct [37].

Accordingly, discriminant validity was examined using the Fornell–Larcker criterion in this study. As shown in Table 3, the square roots of AVE (reported on the diagonal) for all constructs exceed the corresponding inter-construct correlations, providing evidence of adequate discriminant validity among the latent variables. These results support the conceptual distinctiveness of the constructs included in the research model and confirm the suitability of the measurement model for subsequent structural model analysis.

**Table 3:** Discriminant validity assessment using the Fornell–Larcker criterion

Construct	BC	LOC	OSI	SWB	TSCX	TSIV	TSOL	Technostress	TE
BC	0.850								
LOC	0.530	0.825							
OSI	0.356	0.504	0.842						
SWB	0.483	0.480	0.507	0.901					
TSCX	-0.165	-0.205	-0.195	-0.239	0.905				
TSIV	-0.020	-0.242	-0.215	-0.223	0.612	0.873			
TSOL	-0.297	-0.383	-0.238	-0.351	0.506	0.616	0.800		
Technostress	-0.187	-0.329	-0.254	-0.313	0.823	0.875	0.836	0.839	
TE	0.024	-0.186	-0.136	-0.196	0.286	0.368	0.228	0.343	0.797

Note: Diagonal elements (in bold) represent the square roots of AVE. BC = Boundary Control; LOC = Locus of Control; OSI = Offline Social Interaction; SWB = Subjective Well-being; TSCX = Techno-complexity; TSIV = Techno-invasion; TSOL = Techno-overload; TE = Telepressure

### Goodness of fit

The overall goodness of fit (GOF) of the conceptual model was assessed using the global fit index proposed by Vinzi et al., [41], which integrates both the measurement model and the structural model. The GOF is calculated as the geometric mean of the average variance extracted (AVE) and the coefficient of determination (*R*²), using the following formula: $\:{\mathrm{GOF}}=\sqrt{\stackrel{-}{\mathrm{AVE}}}\:x\:\sqrt{\stackrel{-}{{R}^{2}}}$. According to Vinzi et al., [41], GOF values of 0.10, 0.25, and 0.36 indicate small, medium, and large model fit, respectively. In the present study, the average AVE was 0.721, and the average R² was 0.209. Substituting these values into the formula yields:

$\:\mathrm{GOF}=\sqrt{0.721\times\:0.209}=0.388$The resulting GOF value of 0.388 exceeds the threshold for a large effect size, indicating a high level of overall model fit. This result suggests that the proposed model demonstrates satisfactory explanatory power and provides an adequate representation of the relationships among the constructs.

### Path analysis

**Fig. 2:**
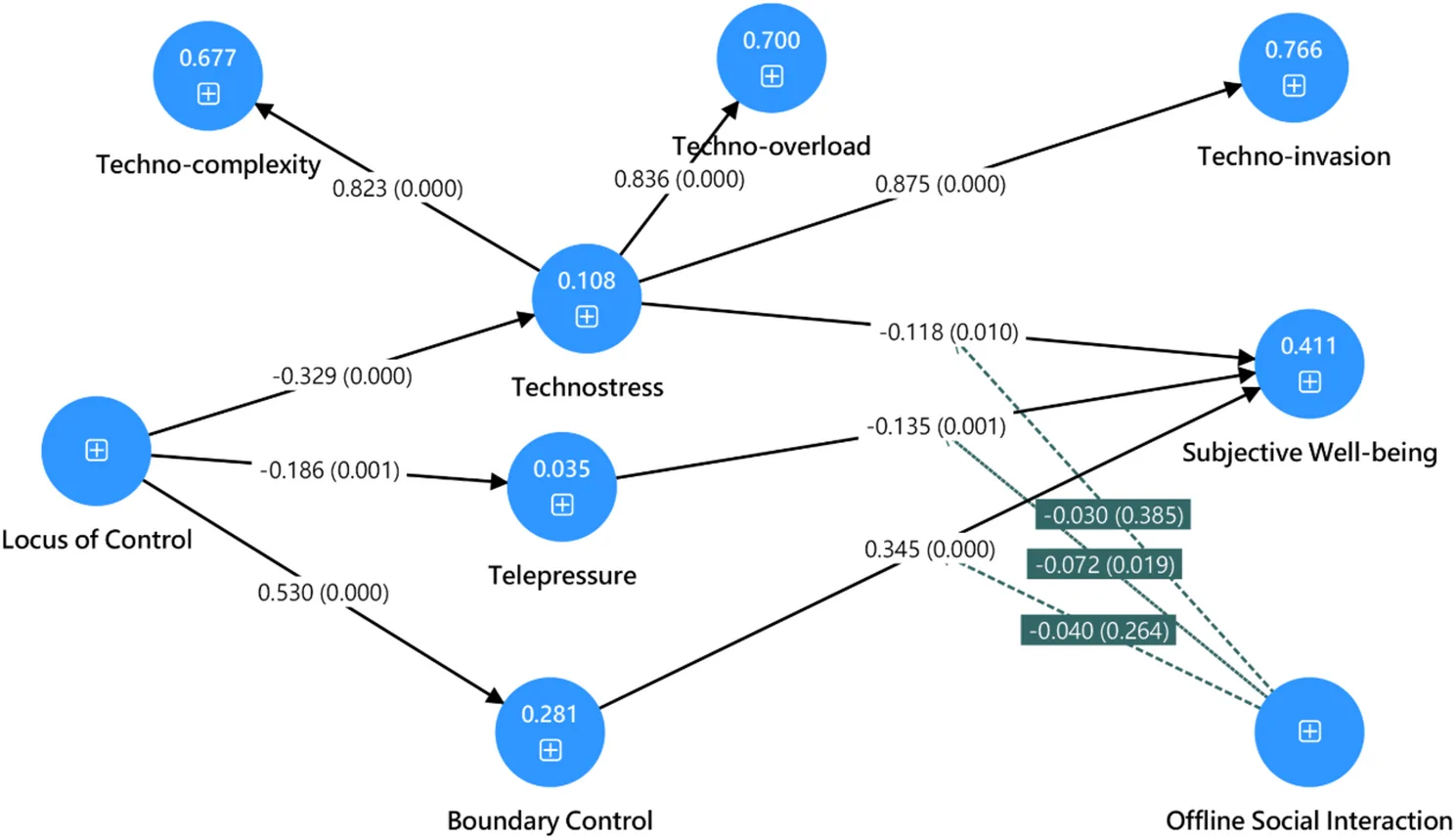
PLS-SEM structural model with path coefficients

Table 4; Fig. 2 report the results of the structural model analysis estimated using Partial Least Squares Structural Equation Modeling (PLS-SEM) with bootstrapping procedures, which are recommended for testing the significance of path coefficients without imposing distributional assumptions [37]. The results indicate that locus of control exerted a significant positive effect on boundary control (β = 0.530, *t* = 13.379, *p* < 0.001), explaining 28.1% of the variance in boundary control (*R*² = 0.281). In addition, locus of control showed significant negative effects on technostress (β = −0.329, *t* = 5.909, *p* < 0.001; *R*² = 0.108) and telepressure (β = −0.186, *t* = 3.441, *p* = 0.001; *R*² = 0.035). These findings suggest that individuals with a stronger internal locus of control experience lower levels of technology-related stress and constant connectivity pressure. Regarding subjective well-being, technostress exhibited a significant negative effect (β = −0.118, *t* = 2.569, *p* = 0.010), whereas boundary control (β = 0.345, *t* = 7.702, *p* < 0.001) and offline social interaction (β = 0.332, *t* = 6.561, *p* < 0.001) demonstrated significant positive effects. Telepressure was also negatively associated with subjective well-being (β = −0.135, *t* = 3.296, *p* = 0.001).

Consistent with established SEM guidelines, the explanatory power of the structural model was evaluated using the coefficient of determination (*R*²), with values around 0.25 and 0.50 indicating weak to moderate explanatory power [37]. Collectively, the predictors accounted for 41.1% of the variance in subjective well-being (*R*² = 0.411), indicating substantial explanatory power of the proposed structural model.

**Table 4:** Structural model path analysis results

Path	Original sample	Standard deviation	t statistics	*p* values	*R* ^2^
Locus of Control → Boundary Control	0.530	0.040	13.379	0.000	0.281
Locus of Control → Technostress	-0.329	0.056	5.909	0.000	0.108
Locus of Control → Telepressure	-0.186	0.054	3.441	0.001	0.035
Technostress → Subjective Well-being	-0.118	0.046	2.569	0.010	0.411
Boundary Control → Subjective Well-being	0.345	0.045	7.702	0.000	
Offline Social Interaction → Subjective Well-being	0.332	0.051	6.561	0.000	
Telepressure → Subjective Well-being	-0.135	0.041	3.296	0.001	

### Mediation effects

Table 5 reports the results of the mediation analysis examining the indirect effects of locus of control on subjective well-being through boundary control, telepressure, and technostress. In line with methodological recommendations for Partial Least Squares Structural Equation Modeling (PLS-SEM), mediation effects were assessed using bootstrapping procedures, with statistical significance determined by *t* values and bias-corrected confidence intervals that do not include zero [37].

The results indicate that boundary control significantly mediates the relationship between locus of control and subjective well-being (β = 0.183, *t* = 6.586, *p* < 0.001). This finding suggests that individuals with a stronger internal locus of control are more likely to establish effective boundary control, which in turn enhances their subjective well-being. In addition, both telepressure (β = 0.025, *t* = 2.230, *p* = 0.026) and technostress (β = 0.039, *t* = 2.279, *p* = 0.023) were found to serve as significant mediators. These results indicate that locus of control indirectly influences subjective well-being by shaping individuals’ experiences of technology-related pressure and stress.

These findings are consistent with established mediation analysis literature, which emphasizes that indirect effects may occur through multiple psychological or contextual mechanisms operating simultaneously [42, 43]. Moreover, the use of bootstrapping to assess indirect effects has been widely recommended as a robust, distribution-free approach, particularly in complex SEM models [37]. Overall, the mediation results provide strong empirical support for the proposed theoretical framework and further demonstrate the explanatory power of the structural model.

**Table 5:** Mediation analysis results

Mediation Path	Original sample	Standard deviation	t statistics	*p* values	2.50%	97.50%
Locus of Control → Boundary Control → Subjective Well-being	0.183	0.028	6.586	0.000	0.132	0.241
Locus of Control → Telepressure → Subjective Well-being	0.025	0.011	2.230	0.026	0.008	0.052
Locus of Control → Technostress → Subjective Well-being	0.039	0.017	2.279	0.023	0.008	0.076

### Moderation effects

Table 6 presents the results of the moderation analysis examining the role of offline social interaction in the relationships between the independent variables and subjective well-being. Following methodological guidelines for partial least squares structural equation modeling, moderating effects were assessed through interaction terms using bootstrapping procedures to determine statistical significance [37]. The results indicate that the interaction between offline social interaction and boundary control was not statistically significant (β = -0.040, *t* = 1.117, *p* = 0.264), nor was the interaction between offline social interaction and technostress (β = -0.030, *t* = 0.868, *p* = 0.385), suggesting that offline social interaction does not uniformly moderate all relationships associated with subjective well-being.

**Fig. 3:**
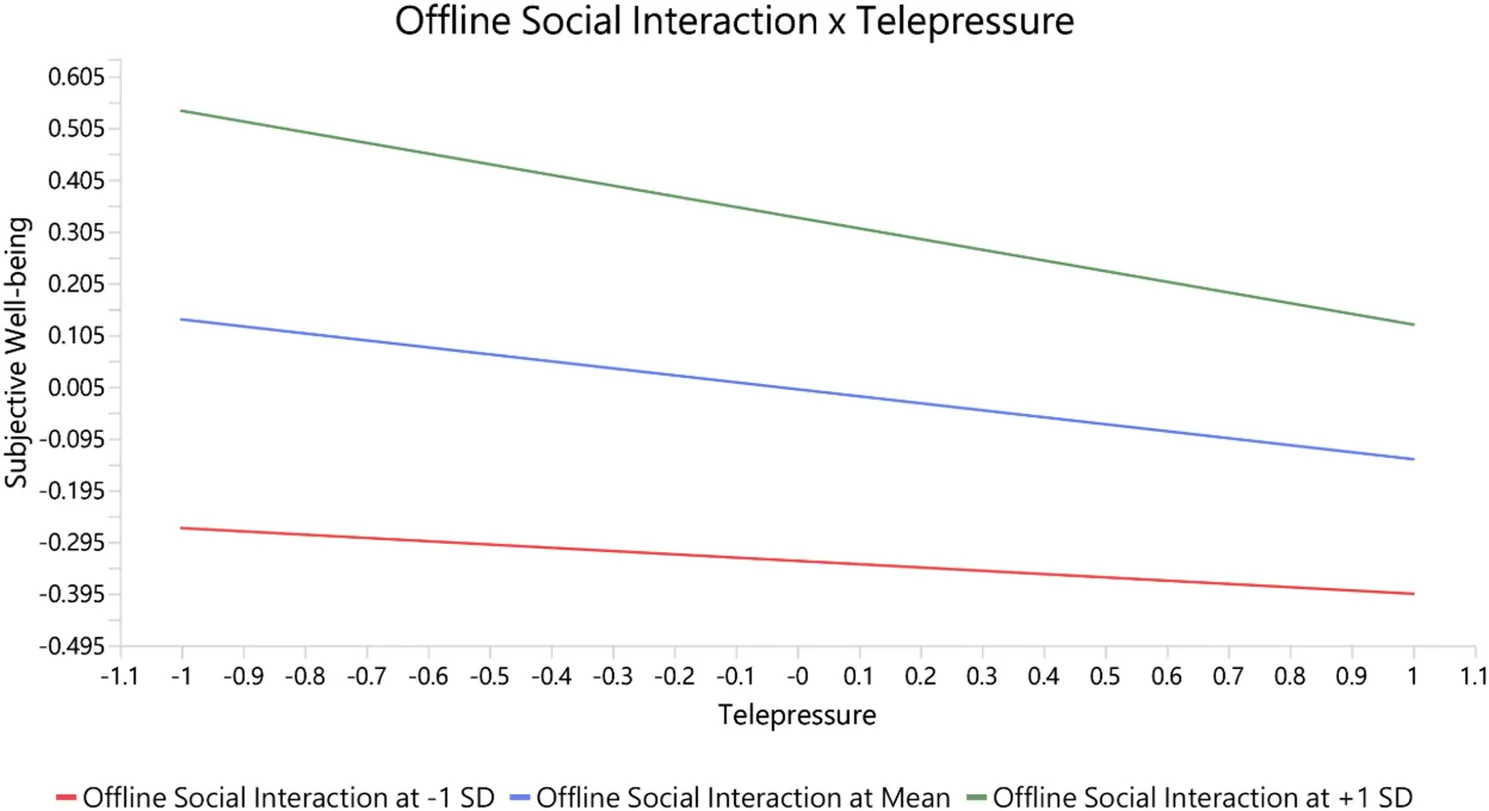
Offline social interaction × telepressure → subjective well-being

In contrast, the interaction between offline social interaction and telepressure was statistically significant (β = -0.072, *t* = 2.346, *p* = 0.019), indicating that offline social interaction moderates the relationship between telepressure and subjective well-being. Specifically, higher levels of offline social interaction attenuate the negative effect of telepressure on subjective well-being. The corresponding interaction pattern is plotted in Fig. 3, which is positioned immediately below Table 6 at the end of this subsection. This pattern of results is consistent with prior methodological research on moderation analysis, which emphasizes that moderating effects are context-specific and may emerge only for particular stressors or relational mechanisms rather than across all hypothesized paths. Overall, these findings align with established statistical theory and provide support for the interpretability and robustness of the proposed structural model.

**Table 6:** Moderation analysis results

Moderation Path	Original sample	Standard deviation	t statistics	*p* values
Offline Social Interaction × Boundary Control → Subjective Well-being	-0.040	0.036	1.117	0.264
Offline Social Interaction × Technostress → Subjective Well-being	-0.030	0.035	0.868	0.385
Offline Social Interaction × Telepressure → Subjective Well-being	-0.072	0.031	2.346	0.019

## Discussion and conclusion

### Overall discussion of key findings

This study investigates the formation of subjective well-being among digital nomads by integrating locus of control, technology-related stressors, boundary control, and offline social interaction within an extreme digital work context. Extending prior research on remote and flexible work, the findings demonstrate that well-being in highly autonomous digital environments is not a direct outcome of flexibility or mobility, but rather emerges from how individuals cognitively interpret digital demands and actively govern their psychological and social boundaries.

First, the results highlight locus of control as an upstream psychological anchor in the digital nomad context. Consistent with prior studies showing that an internal locus of control is associated with lower stress perception and better psychological adjustment in traditional organizational settings [17, 18], this study further demonstrates that control beliefs play an even more critical role when formal organizational structures and supervisory support are largely absent. Unlike conventional employees, digital nomads must independently regulate work rhythms, availability norms, and resource allocation. Under such conditions, locus of control does not directly enhance subjective well-being; instead, it systematically shapes how digital demands are translated into either stress or self-regulatory capacity. This finding extends locus of control theory into extreme digital work environments and responds to calls for greater attention to belief-based psychological resources in digital labor research.

Second, this study advances prior research on digital stress by distinguishing technostress and telepressure as two coexisting yet conceptually distinct demand mechanisms. Previous studies have established the negative effects of technostress on employee well-being, primarily focusing on organizational or telework contexts characterized by relatively stable role expectations and institutional support [9, 10]. Building on this literature, the present findings show that in the digital nomad context, technostress reflects a structural and cumulative resource depletion process, whereas telepressure captures an immediate, psychologically internalized obligation to remain constantly responsive. Both mechanisms independently mediate the relationship between locus of control and subjective well-being, indicating that digital stress among digital nomads cannot be adequately explained by technology overload alone, but must also account for the internalization of availability norms.

Third, among all examined mechanisms, boundary control emerged as the most robust predictor of subjective well-being, underscoring its central role in digital autonomy management. Prior boundary theory research has emphasized the importance of role segmentation and integration strategies in flexible work arrangements [13, 14]. The present study extends this perspective by demonstrating that for digital nomads, well-being is not determined by whether boundaries exist, but by whether individuals perceive themselves as capable of actively deciding when, how, and to what extent boundaries are enacted. In this sense, boundary control functions as a form of psychological governance over digital freedom, transforming flexibility from a potential source of strain into a sustainable resource. This finding also provides empirical support for the autonomy paradox [5], suggesting that autonomy enhances well-being only when accompanied by sufficient self-regulatory capacity.

Taken together, the findings of this study delineate a coherent explanatory framework in which subjective well-being among digital nomads emerges from the interplay between belief-based psychological resources, differentiated digital demands, and self-governed boundary management. By situating digital nomadism as an extreme yet theoretically informative work context, this study not only corroborates prior findings from remote work research, but also extends them by revealing how autonomy, stress, and well-being are dynamically reconfigured when organizational scaffolding is minimized.

### Theoretical implications of the multiple mediation results

By adopting a multiple mediation framework, this study elucidates how locus of control influences subjective well-being among digital nomads simultaneously through technostress, telepressure, and boundary control. Rather than relying on a single explanatory pathway, the findings demonstrate that well-being in highly digitalized work contexts emerges from the concurrent operation of demand-reduction and resource-enhancement mechanisms. This pattern is theoretically consistent with both the Job Demands–Resources (JD-R) model and Conservation of Resources (COR) theory, and extends their applicability to extreme forms of digital work.

First, the results provide empirical support for the JD-R model’s dual-process logic by showing that digital work demands and psychological resources operate in parallel. In the present study, technostress and telepressure function as distinct forms of job demands that undermine well-being through resource depletion, whereas boundary control represents a critical psychological resource that enhances well-being. Importantly, locus of control acts as an upstream belief-based factor that simultaneously weakens demand-related stressors and strengthens resource availability. This finding suggests that locus of control does not merely buffer stress reactions, but rather reconfigures the balance between demands and resources, responding to calls in the JD-R literature to better integrate individual belief systems into demand–resource transformation processes.

Second, from a COR perspective, the multiple mediation results reveal differentiated trajectories of resource loss and resource investment among digital nomads. Technostress and telepressure represent persistent threats of resource loss, whereas boundary control constitutes a strategic capacity to protect and accumulate resources. Individuals with a stronger internal locus of control are more likely to invest in boundary governance, thereby reducing technology-related strain and preventing resource loss spirals. This finding deepens COR theory by highlighting the role of belief-based psychological resources in shaping not only stress perception, but also resource investment strategies under conditions of chronic digital demands.

Third, the simultaneous significance of all three mediating pathways indicates that subjective well-being among digital nomads is not driven by a single psychological mechanism, but instead reflects the combined effects of multiple interrelated processes. Notably, boundary control emerged as the strongest mediator, suggesting that in contexts characterized by high autonomy and constant connectivity, the ability to govern availability and role transitions may exert a more substantial influence on well-being than stress reduction alone. This pattern implies that the core challenge of digital work lies not merely in excessive demands, but in the absence of sustainable self-regulatory mechanisms.

### The moderating role of offline social interaction

This study further examined offline social interaction as a contextual moderator shaping how digital work mechanisms translate into subjective well-being. The moderation results reveal a selective buffering pattern: offline social interaction significantly attenuates the negative effect of telepressure on well-being, but does not significantly moderate the effects of technostress or boundary control. This pattern provides important theoretical insights into the situational specificity of social resources in highly digitalized work contexts.

First, the significant moderation of telepressure highlights the role of offline social interaction as an emotional and attentional buffer against psychologically internalized availability demands. Offline social interaction—through face-to-face engagement, emotional resonance, and embodied presence—offers high levels of attentional immersion and role discontinuity. Such interactions facilitate temporary disengagement from digital cues, thereby weakening the internalized pressure to remain constantly responsive. From a JD-R perspective, offline social interaction functions as a contextual resource that specifically counteracts psychologically intrusive demands, rather than structurally embedded workload pressures.

Second, the absence of a moderating effect on technostress suggests that not all digital stressors are equally susceptible to social buffering. Technostress largely arises from structural and cumulative conditions—such as technology overload, complexity, and invasion—that are embedded in work systems and task architectures. These stressors require changes in task design, technology governance, or individual coping strategies, rather than emotional replenishment alone. This distinction aligns with COR theory, which emphasizes that different stressors demand resource-matched interventions, and that emotional support cannot fully compensate for persistent structural demands.

Third, the non-significant moderation of boundary control further underscores the conceptual distinction between contextual resources and self-regulatory capacities. Boundary control represents an internally governed capability to manage role transitions and availability norms. While offline social interaction may provide emotional support, it does not directly enhance an individual’s perceived control over boundaries, which is more strongly shaped by belief systems, habitual practices, and self-regulatory skills.

### Theoretical contributions

This study offers several theoretically meaningful and cumulative contributions to research on digital work, psychological resources, and well-being.

First, this study conceptualizes digital nomads as a theoretically informative form of an *extreme digital work context*, thereby extending prior research that has predominantly focused on organizational employees or conventional remote workers. By demonstrating that well-being mechanisms function differently in such contexts, this study advances digital labor theory by highlighting how psychological processes are reconfigured when organizational structures are minimized.

Second, this study reconceptualizes locus of control as a central belief-based psychological resource in digital work contexts, rather than a background personality trait. While previous research has primarily treated locus of control as an antecedent of stress perception or psychological health, the present findings show that locus of control systematically shapes both technology-related stressors and boundary governance, thereby exerting indirect effects on subjective well-being through multiple mediating mechanisms.

Third, this study advances digital stress theory by distinguishing technostress and telepressure as two conceptually distinct yet coexisting forms of digital work demands. Whereas prior research has often treated digital stress as a unitary construct, the present findings reveal that technostress primarily reflects structural and cumulative resource depletion, while telepressure arises from the internalization of constant availability expectations.

Fourth, this study highlights boundary control as a core governance mechanism of well-being in highly digitalized and weakly structured work environments, thereby extending the theoretical implications of the autonomy paradox. The findings show that boundary control not only directly enhances subjective well-being, but also serves as a dominant mediator linking locus of control to well-being outcomes. This suggests that autonomy alone does not guarantee well-being; rather, the capacity to *govern* autonomy determines whether digital flexibility becomes a sustainable psychological resource or a source of strain.

Fifth, this study refines social support theory in digital work contexts by revealing the selective moderating role of offline social interaction. Contrary to assumptions that social resources function as universal buffers, the findings indicate that offline social interaction specifically mitigates psychologically internalized stressors such as telepressure, while exerting no significant moderating effect on structurally embedded technostress or internally governed boundary control. This contribution underscores the mechanism-dependent nature of social resources and encourages more precise theorization of when and how social support operates in digitalized work environments.

### Practical implications

Beyond its theoretical contributions, this study offers several actionable practical implications for digital work platforms, digital nomads themselves, and policymakers seeking to promote sustainable well-being in highly digitalized work environments.

#### Implications for digital work platforms and technology designers

The findings suggest that digital work platforms play a critical role in shaping users’ experiences of telepressure and technostress, particularly through implicit norms surrounding responsiveness and availability. Platforms that prioritize immediacy—such as real-time notifications, visible response indicators, or algorithmic rewards for rapid replies—may inadvertently intensify telepressure and undermine users’ well-being.

Accordingly, platform designers are encouraged to normalize asynchronous communication by offering default delay options, clearer availability status settings, and system-level signals that legitimize non-immediate responses. In addition, providing customizable notification controls and boundary-setting tools may help users exercise greater boundary control, thereby transforming digital flexibility into a sustainable resource rather than a source of strain. These design choices are especially important for digital nomads, who lack organizational buffers and must rely heavily on platform architectures to regulate work rhythms.

These recommendations must, however, be qualified by the market logic within which much digital nomadic work is transacted. In platform-mediated environments, income continuity depends on visible availability, short response times, and favorable client ratings, and these signals are routinely incorporated into the ranking systems that govern work allocation [26, 27]. De-emphasizing immediacy at the platform level could therefore disadvantage the very workers it is intended to protect, particularly those at an early stage of reputation accumulation who have the least scope to decline work. Recognizing this tension, we advance a differentiated rather than a uniform prescription. Responsiveness norms could be calibrated to task type, distinguishing genuinely time-critical deliverables from work for which urgency is conventional rather than substantive; to client-relationship stage, since established relationships tolerate asynchrony that new engagements do not; and to service category, given that consulting, creative, and technical support work differ markedly in their legitimate response windows. Platforms might further decouple responsiveness from reputation by displaying a worker’s declared availability window alongside their response metrics, so that promptness within stated hours, rather than continuous reachability, becomes the basis of evaluation.

The scope of these recommendations should also be delimited. They apply most directly to digital nomads who hold direct client relationships, retained contracts, or employment arrangements, and least directly to those whose earnings depend on competitive bidding within reputation-ranked marketplaces. For the latter group, platform architecture is unlikely to relieve responsiveness pressure in the near term, and boundary control will remain a resource that individuals must generate themselves. That constraint does not weaken the present findings so much as sharpen their implication: where structural remedies are unavailable, the upstream psychological resource identified here becomes correspondingly more consequential for well-being.

#### Implications for digital nomads and independent remote workers

For digital nomads, the findings underscore that well-being is not guaranteed by mobility or autonomy alone, but depends critically on how autonomy is governed. Individuals with a stronger internal locus of control are better positioned to manage digital demands, not because they experience fewer external pressures, but because they actively shape how those pressures are interpreted and managed.

Practically, this suggests that digital nomads should invest in developing boundary governance skills, such as establishing explicit availability rules, scheduling technology-free periods, and cultivating routines that separate work engagement from recovery time. Importantly, the results also indicate that relying solely on stress avoidance strategies may be insufficient; instead, proactive boundary management represents a more effective pathway to sustaining well-being under conditions of constant connectivity.

#### Implications for policymakers and shared work environments

At the policy level, the findings highlight the importance of recognizing digital nomads as a distinct workforce with unique psychological risks and support needs. Rather than focusing exclusively on digital infrastructure provision (e.g., internet access), policymakers and urban planners should consider how physical environments and community initiatives can foster meaningful offline social interaction.

The selective moderating role of offline social interaction suggests that co-working spaces, community hubs, and local programs that facilitate face-to-face engagement may serve as protective resources against psychologically internalized pressures such as telepressure. However, such initiatives should be understood as complementary rather than substitutive to structural interventions targeting technostress and boundary governance. Policies that integrate digital flexibility with opportunities for social embedding are more likely to support long-term psychological sustainability among mobile digital workers.

### Limitations and future research directions

Several limitations of this study should be acknowledged, which also point to directions for future research. First, the use of a cross-sectional design restricts causal interpretation. Future studies may adopt longitudinal or experience-sampling approaches to capture dynamic changes in digital stress, boundary control, and well-being over time. Second, the reliance on self-reported data may introduce subjective bias. Future research could incorporate behavioral or objective indicators, such as digital communication logs or response patterns, to complement self-report measures and enhance measurement validity. Third, although digital nomads were examined as a theoretically informative group, contextual differences such as cultural or institutional environments were not explicitly considered. Comparative or cross-cultural studies would help assess the generalizability of the proposed mechanisms. Finally, while this study focuses on locus of control as a core psychological resource, future research could extend the model by examining additional individual resources, such as self-regulation capacity or digital literacy, and by exploring broader outcomes related to long-term sustainability in digital work.

Three further limitations warrant explicit acknowledgment. First, participants were recruited by convenience sampling through remote work communities, digital nomad forums, and social media groups. Self-selection into these channels plausibly over-represents nomads who are socially connected and digitally engaged, and the sample cannot be treated as probabilistically representative of the population; the moderating effect of offline social interaction, in particular, may be estimated on a restricted range. Second, digital nomad status was established through platform-administered screening whose operational criteria were not disclosed to the research team, together with respondents’ self-reported work characteristics, and the boundary between digital nomads, long-term remote employees, and mobile freelancers is empirically porous. Some respondents may therefore correspond only partially to the theoretical construct on which the contrast developed in Sect.  2.1 rests. Future studies should apply verifiable screening criteria, such as documented cross-border mobility or platform work histories, and report the proportion of respondents satisfying each criterion. Third, because all constructs were measured by self-report within a single instrument administered at one point in time, common method bias cannot be ruled out, and observed relationships may be inflated relative to their true magnitude. Subsequent research should adopt procedural and statistical remedies, including temporal separation of predictor and criterion measurement, marker-variable techniques, and the incorporation of multi-source or behavioral data.

## Supplementary information

Below is the link to the electronic supplementary material.

Supplementary Material 1

## Data Availability

The datasets generated and/or analyzed during the current study are available in the Zenodo repository: https://doi.org/10.5281/zenodo.18267013.
