# Supplementary material for: How locus of control shapes digital nomads’ subjective well-being: the mediating roles of telepressure, technostress, and boundary control
Source: BMC Psychol. 2026 Sep 26;14:1622. doi: 10.1186/s40359-026-05635-7 (PMC13749687; doi:10.1186/s40359-026-05635-7)
Supplement: Supplementary file 1 [file 40359_2026_5635_MOESM1_ESM.docx]

Supplementary Material 1

Survey Questionnaire: Locus of Control and Subjective Well-Being among Digital Nomads

(1) Introduction

Thank you for participating in this research. This survey aims to examine how locus of control influences subjective well-being among digital nomads through telepressure, technostress, and boundary control, as well as the moderating role of offline social interaction. All responses are anonymous and will be used solely for academic research purposes. Completing this questionnaire takes approximately 15–20 minutes. Unless otherwise stated, all items are measured using a seven-point Likert scale:

1 = Strongly disagree
2 = Disagree
3 = Somewhat disagree
4 = Neutral
5 = Somewhat agree
6 = Agree
7 = Strongly agree

(2) Scale Sources

The measurement items were adapted from established scales in the literature.

Locus of Control: Rotter (1966)

Telepressure: Barber & Santuzzi (2015)

Technostress: Tarafdar et al. (2007)

Boundary Control: Kossek et al. (2012)

Subjective Well-Being: Diener et al. (1999) and WHO-5 Well-Being Index

Part 1. Demographic Information

Gender

□ Male
□ Female

Age

□ 18–24
□ 25–29
□ 30–34
□ 35–39
□ 40–49
□ 50 or above

Highest level of education

□ High school or below
□ Associate degree
□ Bachelor’s degree
□ Master’s degree or above

Average weekly remote working hours (past month)

□ 0–10 hours
□ 11–19 hours
□ 20–29 hours
□ 30–39 hours
□ 40–49 hours
□ 50 hours or more

Current city of residence

Part 2. Locus of Control (LOC)

LOC1: I can influence my work outcomes.

LOC2: Success mainly depends on my effort.

LOC3: I can control important work decisions.

LOC4: I can effectively change unsatisfactory work situations.

LOC5: I can actively create opportunities in my favor.

LOC6: My efforts can bring positive results at work.

Part 3. Telepressure (TE)

TE1: It is difficult for me to focus on other things when I receive a message from someone.

TE2: I can concentrate better on other tasks once I have responded to my messages.

TE3: I cannot stop thinking about a message until I have responded.

TE4: I feel a strong need to respond to others immediately.

TE5: I have an overwhelming feeling to respond right away when I receive a request from someone.

TE6: It is difficult for me to resist responding to messages immediately.

Part 4. Technostress (TS)

Techno-overload

TSOL1: Technology requires me to do more work in less time.

TSOL2: I handle too many digital tasks simultaneously.

TSOL3: The volume of messages often overwhelms me.

Techno-invasion

TSIV1: Technology brings work into my private life.

TSIV2: Even when I am on vacation, I still feel tied to technology.

TSIV3: I find it difficult to disconnect from devices and digital platforms.

Techno-complexity

TSCX1: New technological tools often confuse me.

TSCX2: Keeping up with new technologies requires a lot of time.

TSCX3: I need to invest extra effort to use new systems or tools effectively.

Part 5. Boundary Control (BC)

BC1: I can decide when to deal with work-related messages.

BC2: I can choose not to let work interfere with my personal time.

BC3: I can set clear boundaries between work and personal life.

BC4: When necessary, I can turn off notifications and communication channels.

Part 6. Offline Social Interaction (OSI)

OSI1: I frequently have face-to-face interactions with friends or colleagues.

OSI2: I often participate in in-person social or professional gatherings.

OSI3: I receive emotional support through face-to-face interactions.

OSI4: In-person interactions make me feel connected and accepted.

Part 7. Subjective Well-Being (SWB)

SWB1: I feel cheerful and in good spirits.

SWB2: I feel calm and relaxed.

SWB3: I feel energetic and full of vitality.

SWB4: I feel well-rested when I wake up.

SWB5: I feel interested in my daily activities.

Closing Statement

Thank you for completing this questionnaire. Your responses are extremely valuable for advancing research on digital work and well-being.
